# Advanced adenoid cystic carcinoma (ACC) is featured by SWI/SNF chromatin remodeling complex aberrations

**DOI:** 10.1007/s00432-018-2783-5

**Published:** 2018-10-31

**Authors:** Beata Jagielska, Elzbieta Sarnowska, Nataliia Rusetska, Iga Jancewicz, Monika Durzynska, Szymon Kubala, Ewa Chmielik, Piotr Paul, Tomasz Rutkowski, Tomasz J. Sarnowski, Janusz A. Siedlecki

**Affiliations:** 1 Department of Oncology and Internal Medicine, Marie Sklodowska-Curie Memorial Cancer Center, Institute of Oncology, Warsaw, Poland; 2 Department of Molecular and Translational Oncology, Marie Sklodowska-Curie Memorial Cancer Center, Institute of Oncology, Warsaw, Poland; 3 Department of Pathology, Marie Sklodowska-Curie Memorial Cancer Center and Institute of Oncology, Warsaw, Poland; 4 Institute of Biochemistry and Biophysics, Polish Academy of Sciences, Warsaw, Poland; 5 Department of Pathology, Marie Sklodowska-Curie Memorial Cancer Center and Institute of Oncology, Gliwice, Poland; 6 Inpatient Department of Radiation and Clinical Oncology, Marie Sklodowska-Curie Memorial Cancer Center, Institute of Oncology, Gliwice, Poland

**Keywords:** Adenoid cystic carcinoma, SWI/SNF complex, Androgen receptor (AR), Progressive disease, Chemoresistance

## Abstract

**Purpose:**

Adenoid cystic carcinoma (ACC) is a rare neurotropic cancer with slow progression occurring in salivary glands and less frequently in other body parts. ACC is featured by hyperchromatic nuclei and various mutations in genes encoding chromatin-related machineries. The ACC treatment is mainly limited to the radical surgery and radiotherapy while the chemotherapy remains ineffective. As the knowledge about molecular basis of ACC development is limited, we investigated here the molecular features of this disease.

**Patients and methods:**

This study included 50 patients with ACC. Transcript profiling of available ACC samples vs normal salivary gland tissue, quantitative real-time PCR (qRT-PCR) transcript level measurements and the immunohistochemistry (IHC) for SWI/SNF chromatin remodeling complex (CRC) subunits and androgen receptor on surgery-derived paraffin-embedded samples were performed.

**Results:**

Transcriptomic study followed by Gene Ontology classification indicated alteration of chromatin-related processes, including downregulated transcript levels of main SWI/SNF CRC subunits and elevated expression of BRM ATPase-coding S*MARCA2* gene in ACC. Subsequent IHC indicated broad accumulation of BRM ATPase and several SWI/SNF subunits, suggesting affected control of their protein level in ACC. The IHC revealed ectopic, heterogeneous expression of androgen receptor (AR) in some ACC cells.

**Conclusions:**

Our study indicated that ACC features aberrant expression of genes controlling chromatin status and structure. We found that the balance between SWI/SNF classes is moved towards the BRM ATPase-containing complex in ACC. As BRM is known to be involved in chemoresistance in cancer cells, this observation may be the likely explanation for ACC chemoresistance.

**Electronic supplementary material:**

The online version of this article (10.1007/s00432-018-2783-5) contains supplementary material, which is available to authorized users.

## Introduction

Adenoid cystic carcinoma (ACC) is an uncommon malignancy. Primary lesions usually arise from salivary glands, more frequently from minor than major ones. Other localizations include paranasal sinuses, larynx, lacrimal glands, bronchi, mammary and skin (Coca-Pelaz et al. 2015; Dillon et al. 2016). This type of cancer most frequently occurs between the fourth and the sixth decades of life. ACC is rather indolent disease with slow growth and late development of distant metastases, but it may also be fast-growing malignancy with early distant spread. When diagnosed at an early stage, it usually appears as a single and oval lesion (usually with smaller than 6 cm diameter) well defined and partially capsulated with a pronounced tendency to aggressively infiltrate surrounding tissues along nervous tissue structures. Metastases to regional lymph nodes and distant sites occur, and ACC recurrences may appear many years after initial treatment (van der Wal et al. 2002; Kokemueller et al. 2004) although the lymph node metastases are uncommon (Ferlito 2016; He et al. 2017). Treatment of these type of cancer remains limited to surgery with or without adjuvant radiotherapy (proton beam) (Gentile et al. 2017). Moreover, no systemic treatment has been found to be effective (Laurie et al. 2011; Xu et al. 2018a). Due to peculiar biology of this malignancy, various attempts have been made including the use of metronomic chemotherapy in patients with advanced or disseminated disease (Visa et al. 2010; Caballero et al. 2013; Cassidy et al. 2018).

The ACC mutational landscape was determined and revealed a low exonic somatic mutation rate and wide mutational diversity. Among others, mutations in genes involved in histone acetylation/deacetylation (*ARID4B, ARID5B, BRD1, FTSDJ1, MLL3*), as well as encoding histones (*HIST1H2AL, HIST1H1E*), were found suggesting that chromatin dysregulation may promote ACC development (Ho et al. 2013). Additionally, in ACC, mutations in *SMARCA2*, a gene encoding the BRM ATPase of the SWI/SNF chromatin remodeling complex (CRC), were found. Moreover, 2% of ACC cases are featured by mutations in *ARID1A* and *SMARCE1* genes, coding for BAF250a and BAF57 non-core SWI/SNF CRC subunits, respectively, consistently suggesting aberrant SWI/SNF CRC function in ACC (Ho et al. 2013).

In this study, we attempted to better understand the molecular features of adenoid cystic carcinoma. The re-analysis of available transcriptomic data followed by gene ontology (GO) classification indicated that ACC is featured by the upregulation of steroid hormone receptor activity, carbohydrate metabolism, cell proliferation and drug transport-related processes. By contrast, the downregulated processes were mostly related in ACC to the control of chromatin structure and function including chromatin remodeling and organization, histone deacetylase and acetyltransferase complex, telomere maintenance and methyltransferase activity. Among downregulated genes, we found genes encoding nearly all subunits of SWI/SNF chromatin remodeling complex. The subsequent immunohistochemistry (IHC) staining showed alterations in SWI/SNF CRC subunits’ abundance and ectopic expression of androgen receptor in ACC compared to margin tissue. Among BRM-target genes overexpressed in ACC, a class of genes involved in chemoresistance was identified. Taking together, our findings suggest an important role of SWI/SNF CRC impairment in the adenoid cystic carcinoma development and provided a likely explanation of the ACC resistance to various specific chemotherapy treatments.

## Materials and methods

### Patients’ characteristics

A group of 50 patients from, Marie Sklodowska-Curie Memorial Cancer Center and Institute of Oncology in Warsaw and Gliwice with diagnosed adenoid cystic carcinoma was subjected to this study. Primary lesions were most often localized in submandibular and parotid glands (Table 1). All 50 patients underwent primary surgical treatment. The patients represent whole area of Poland.

**Table 1:** List of the most important patient characteristics in a group consisting of 50 ACC patients

Characteristic	Number of patients (*n* = 50)
Gender	
Female	25 (50%)
Male	25 (50%)
Age	
Mean age	53 (min. 22, max. 90)
< 60	33 (66%)
≥ 60	17 (34%)
Localization	
Minor salivary gland	31 (62%)
Major salivary gland	19 (38%)
pT	
pT1	6 (12%)
pT2	19 (38%)
pT3	3 (6%)
pT4	11 (22%)
n/a	11 (22%)
Treatment	
Radiotherapy	45 (90%)
Chemotherapy	6 (12%)
Progression	
Total number	14 (28%)
Female	5 (36%)
Male	9 (64%)

### Microarray re-analysis

Microarray datasets were obtained from Gene Expression Omnibus (GEO) database with the accession number GSE28996 for ACC samples and GSE36820 for normal salivary gland (Moskaluk et al. 2011). The nine ACC and three healthy salivary gland corresponding sample data files were re-analyzed using GeneSpring GX (Agilent) software according to advanced guided workflow.

### Immunohistochemistry

Immunohistochemical staining was performed on 4-µm FFPE tumor sections using the EnVision FLEX+, Mouse, High-pH Detection System (Dako, Glostrup, Denmark). Sections were deparaffinized with xylene and rehydrated in ethanol solutions. Heat-induced epitope retrieval was carried out in Target Retrieval Solution (Dako) for 20 min at 96 °C. After cooling, the slides were treated for 5 min with an endogenous peroxidase blocker (Dako), incubated with monoclonal antibody against androgen receptor (D6F11, Cell Signaling), BRG1, INI1, BAF155, BAF170, BAF250a and BRM (P680, D9C2, D7F8S, D8O9V, D2A8U, D9E8B, Cell Signaling) for 30 min at room temperature, and then labeled with the EnVision FLEX+, Mouse, High-pH Detection System (Dako). The color reaction product was developed with 3,3′-diaminobenzidine tetrahydrochloride (Dako) as a substrate, and nuclear contrast was achieved with hematoxylin counterstaining.

### Transcript level measurements

RNA was extracted from paraffin-embedded samples using RecoverAll™ Total Nucleic Acid Isolation Kit for FFPE (Invitrogen). Contamination with genomic DNA was removed using DNAse I treatment according to the manufacturer’s protocol. Aboult 300 ng of total RNA was used for the first-strand cDNA synthesis using Transcriptor First Strand Synthesis kit (ROCHE) and oligo-dT primer and random hexamer mix. Transcript levels of studied genes were measured in triplicates using Applied Biosystems 7500 Fast device with specific primers and SybrGreen. For *SMARCA2*, following primers were used hBRMq2F: TCCGAGGCAAAATCAGTCAAG and hBRMq2R: TTCCTCGATTTGGCCTTTTCT. As the reference primers UBI1: ATTTGGGTCGCGGTTCTTG and UBI2: TGCCTTGACATTCTCGATGGT specific for *UBI (UBIQUITIN*) gene were used. For the relative expression level calculation, the 2^−ΔΔCt^ method was used.

### Statistical methods

For statistical analysis, MedCalc software and GraphPad Prism 5.0 with Shapiro–Wilk normality test, Mann–Whitney rank test for independent sample and paired *t* test were used. *p* value < 0.05 was considered to be statistically significant.

## Results

### Gene expression profiling in ACC

**Fig. 1:**
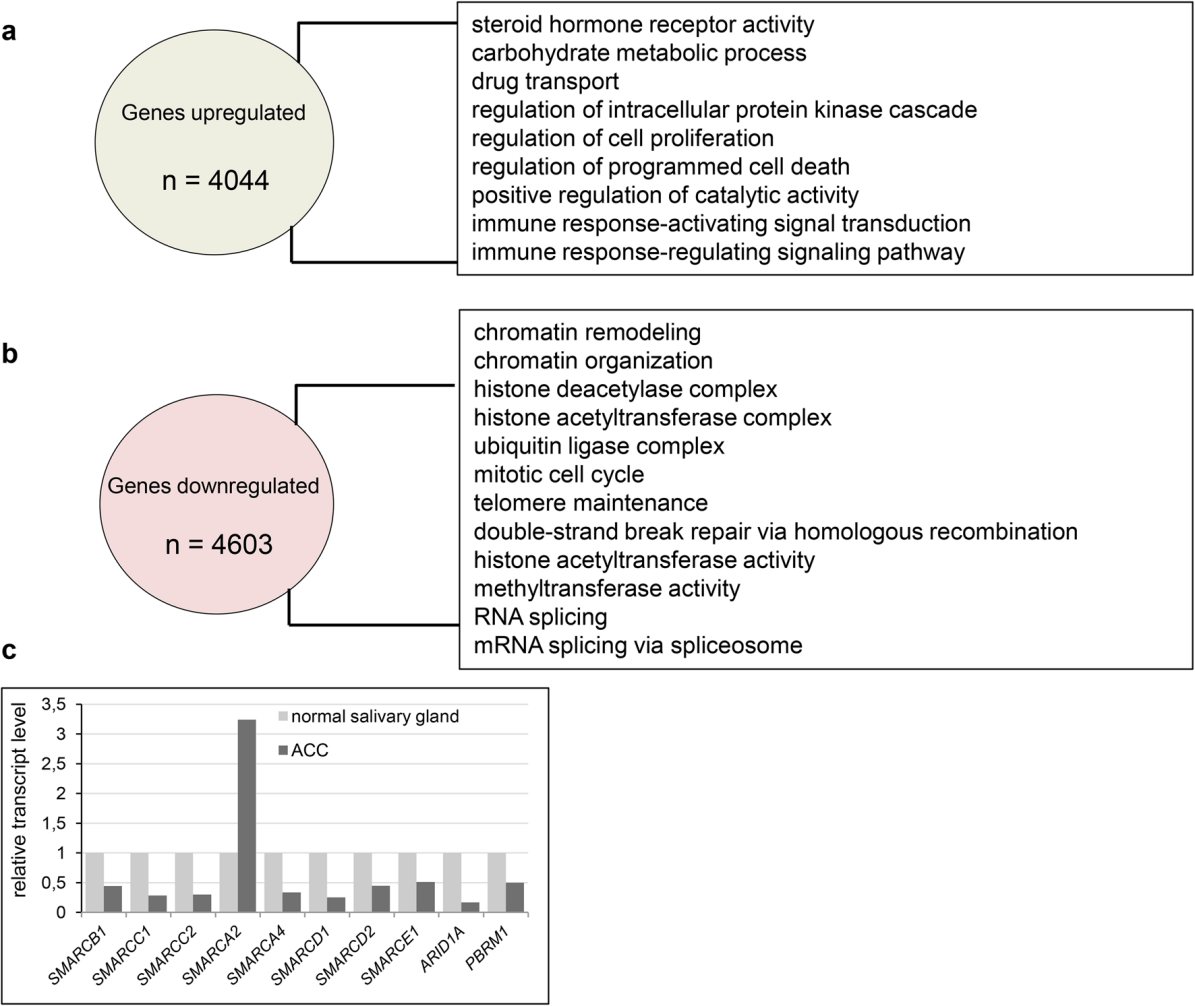
Adenoid cystic carcinoma is featured by transcriptomic changes affecting various regulatory pathways. **a** Genes upregulated in ACC patient samples compared to normal salivary gland and GO term enrichment among identified genes. **b** Genes downregulated in ACC patient samples compared to normal salivary gland, GO term enrichment in processes among identified genes. **c** Genes coding for SWI/SNF subunits exhibit altered transcript level in ACC samples compared to normal salivary gland

The adenoid cystic carcinoma is a severe disease, which is featured by the resistance to various treatments such as chemotherapy (i.e., see Visa et al. 2010; Caballero et al. 2013; Cassidy et al. 2018) including metronomic therapy. Given this fact we decided to investigate the transcriptional profiles of ACC samples and compare them with transcript profiles of normal tissue to identify the molecular features which could be helpful in the explanation of such severe chemoresistancy of ACC. The reanalysis of 9 ACC vs 3 normal salivary gland samples derived from GEO database (accession number GSE28996 and GSE36820) resulted in the identification of 8647 differentially expressed genes. 4603 genes were downregulated in ACC samples compared to healthy control while 4044 genes exhibited elevated expression. Gene Ontology analysis showed enrichment of steroid hormone receptor activity, carbohydrate metabolism, cell proliferation and drug transport (Fig. 1a, Supplementary Table 1 Sub-table 1) as the most common GO processes upregulated in ACC. By contrast, processes downregulated in ACC were mostly related to the control of chromatin structure and functions such as chromatin remodeling, chromatin organization, histone deacetylase and acetyltransferase complex, telomere maintenance and methyltransferase activity as well as RNA splicing and mRNA splicing via spliceosome (Fig. 1b, Supplementary Table 1 Sub-table 2).

### Aberrations of SWI/SNF complex in ACC

**Fig. 2:**
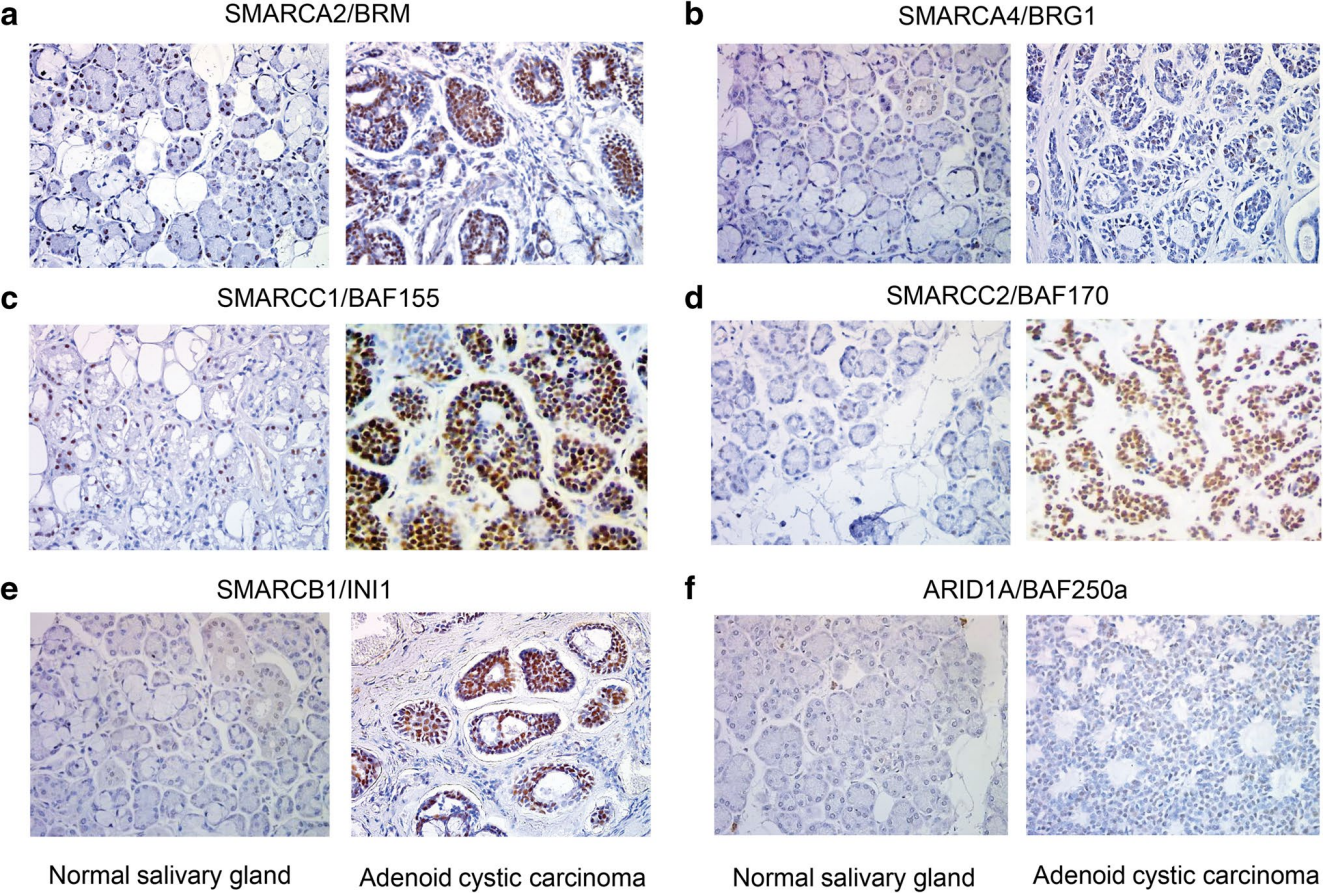
ACC cells are featured by altered abundance of SWI/SNF core and non-core subunits. IHC staining for **a** BRM. **b** BRG1. **c** BAF155. **d** BAF170. **e** INI1. **f** BAF250a in ACC samples compared to normal salivary gland tissue (**a**–**f** magnification: × 40)

In ACC, among the ‘chromatin remodeling’ GO class the following downregulated genes coding for various SWI/SNF subunits were found: *SMARCB1, SMARCC1, SMARCC2* and *SMARCA4* (coding for SWI/SNF core subunits) and *ARID1A, PBRM1, SMARCD1, SMARCD2* and *SMARCE1* (encoding non-core SWI/SNF subunits). Interestingly, the transcript level of *SMARCA2* gene coding for the BRM ATPase subunit of SWI/SNF CRCs was significantly elevated compared to the control tissue (Fig. 1c). Therefore, we employed immunohistochemistry (IHC) to investigate the protein level of SWI/SNF subunits such as BRM, BRG1, BAF155, BAF170, INI1 and BAF250a in paraffin-embedded ACC and healthy samples. We found that consistently with our transcript profiling analysis the BRM protein level was significantly elevated in cancer cells compared to normal salivary gland tissue (Fig. 2a). Surprisingly, the abundance of the second SWI/SNF ATPase, BRG1, did not show any alterations in the IHC study or was only discretely elevated in some ACC cells (Fig. 2b); however, the BRG1 encoding *SMARCA4* gene exhibited decreased transcript level in ACC samples. Interestingly, the protein levels of BAF155, BAF170 and INI1 were even higher in ACC samples than in healthy salivary gland tissue, although the corresponding genes were downregulated at the transcript level in ACC samples (Fig. 2c–e). Moreover, also for BAF250a the slightly elevated abundance was observed in ACC (Fig. 2f).

**Fig. 3:**
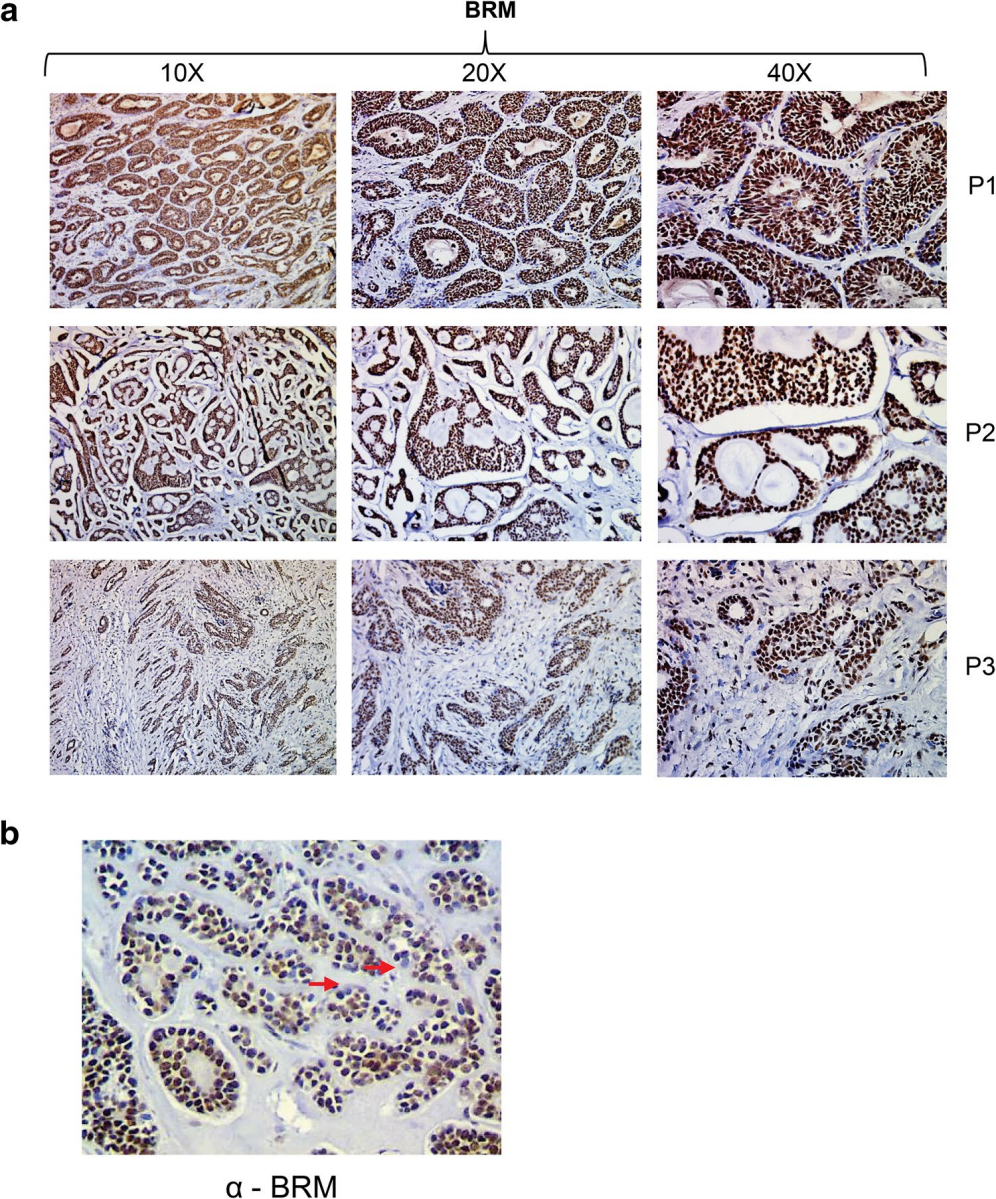
The BRM overaccumulation is a general feature of ACC. **a** Images of ACC samples from three different patients taken under three magnifications showing that strong BRM staining is ubiquitous and not restricted to ACC regions. **b** The BRM-negative staining was limited to sporadically observed single cells but not to ACC regions confirming ubiquitous BRM protein accumulation in ACC

Furthermore, to assess if the BRM protein is ubiquitously accumulated in ACC or only some regions of the tumor are featured by the BRM overexpression we performed careful inspection of all ACC samples under various magnifications (Fig. 3a). We found that ACC is featured by the general BRM over accumulation as in all ACC samples the ACC cells were stained independently on the part of the tumor (Fig. 3a). The unstained cells but not unstained regions were only sporadically observed in the case of BRM staining in ACC samples (Fig. 3b). As we found that ACC, a highly heterogeneous cancer type, is featured by the nearly homogeneous increase of BRM protein abundance we concluded that BRM overaccumulation in most of the ACC cells may have a great impact on the general ACC features.

### The levels of transcripts of genes coding for SWI/SNF subunits vary between patients

**Fig. 4:**
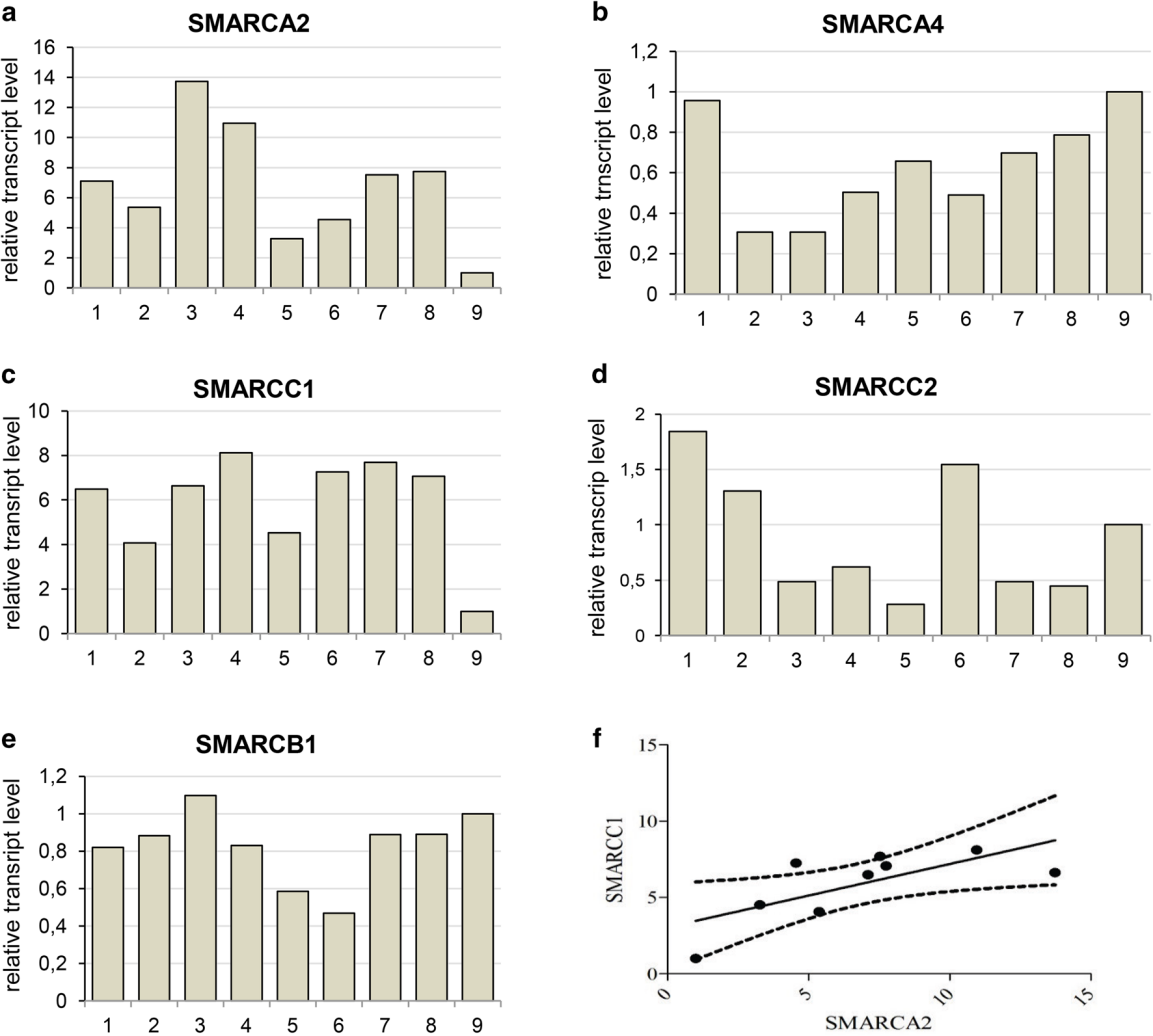
Expression of genes coding for subunits of SWI/SNF CRCs varies between ACC patient-GEO database data reanalyses. **a** SMARCA2-BRM. **b** SMARCA4-BRG1. **c** SMARCC1-BAF155. **d** SMARCC2-BAF170. **e** SMARCB1-INI1-encoding gene transcript level between ACC patients samples. **f** Correlation between SMARCA2 and SMARCC1 *p* value 0.0339, Pearson *r* = 0.7049

**Fig. 5:**
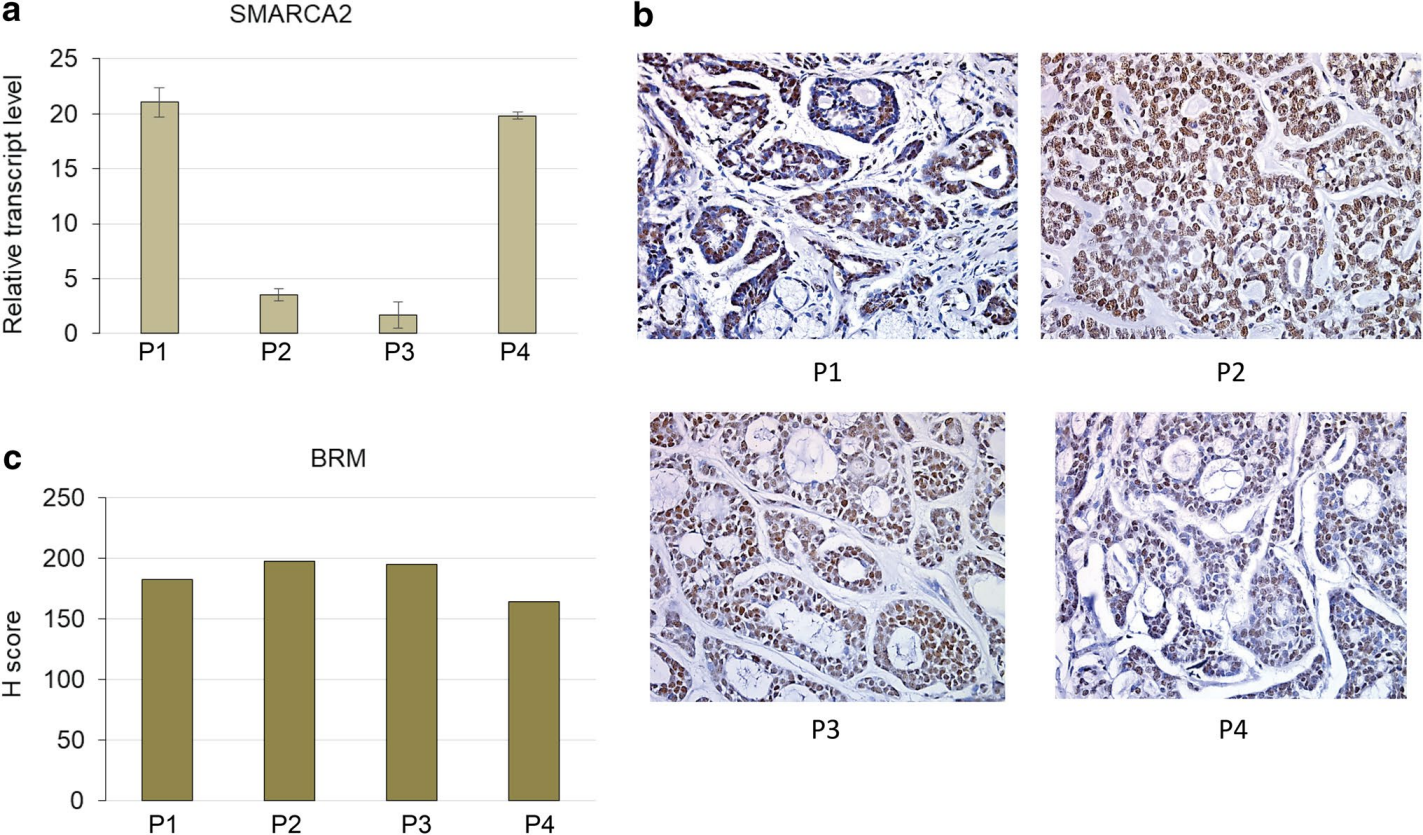
The *SMARCA2* transcript levels vary between particular patients but the BRM staining always shows strong BRM overaccumulation. **a** The relative transcript level of BRM-encoding *SMARCA2* gene varies between patients. **b** The immunohistochemistry staining of BRM protein abundance in the same patients as in **a**. Magnification: × 40. **c** The H score analysis for patients from **a, b**

More detailed analysis indicated that the *SMARCA2* (BRM) gene expression level differs between ACC samples derived from various patients (Fig. 4a). Subsequent analysis of transcript levels of other genes encoding core SWI/SNF subunits such as *SMARCA4*(BRG1), *SMARCC1*(BAF155), *SMARCC2*(BAF170) and *SMARCB1*(INI1) indicated similar-to-*SMARCA2* variability between particular patient samples (Fig. 4b–e). However, the comparative analysis of expression levels of genes encoding core SWI/SNF subunits showed significant positive correlation only for *SMARCA2* and *SMARCC1* genes (Fig. 4f), indicative of the occurrence of potential multiple mechanisms of SWI/SNF CRC impairment including its aberrant stoichiometry in ACC. Moreover, the careful comparison of alterations in the transcript level of BRM-encoding *SMARCA2* gene (Fig. 5a) with the BRM protein abundance indicated that independently on the individual differences in *SMARCA2* gene expression, the BRM protein level was always dramatically elevated suggesting the existence of BRM protein stabilizing mechanism in the ACC cells (Fig. 5b, c).

### Ectopic expression of androgen receptor features some ACC cells

**Fig. 6:**
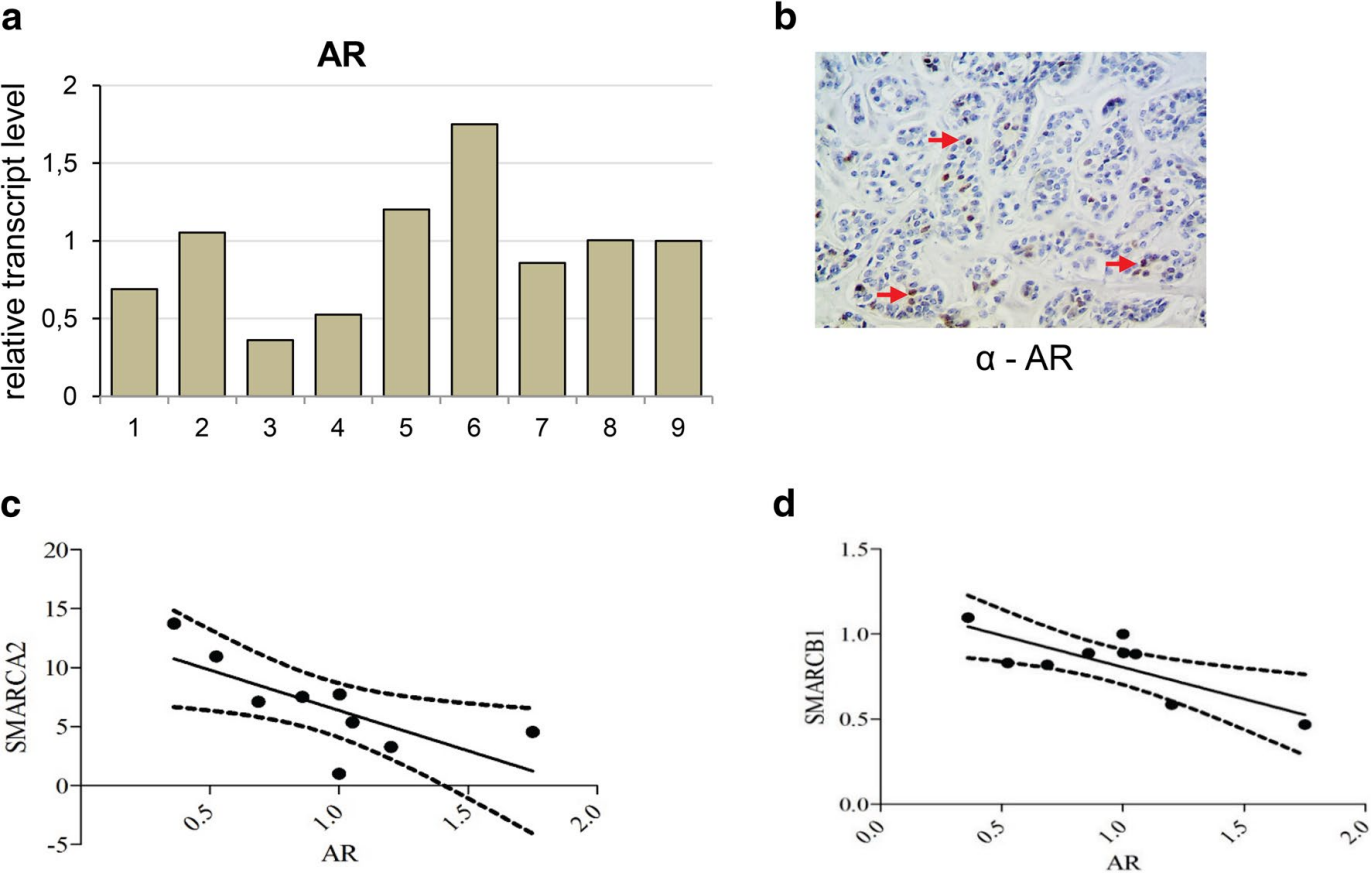
Ectopic expression of androgen receptor features some ACC cells and negatively correlates to *SMARCA2* and *SMARCB1* genes. **a** Androgen receptor (AR) transcript level in carcinoma adenoides cysticum (ACC) patients. **b** Positive staining for AR in some cancer cells (indicated by red arrowhead), magnification × 40. **c** Correlation between SMARCA2 and AR transcript, *p* value 0.0289, Spearman *r* = − 0.7196. **d** Correlation between SMARCB1 and AR *p* value 0.0125, Pearson *r* = − 0.7832

Molecular pathway analysis using GeneSpring GX software showed significant differences in alpha 6 beta 4 integrin pathway known to be involved in epithelial cell migration (Mercurio et al. 2001), EGFR pathway and others mostly immunology-dependent pathways and androgen receptor (AR) pathway (Supplementary Table 2). Therefore, we analyzed the expression of AR in ACC samples (Fig. 6a) and performed the immunohistochemical study on seven paraffin-embedded samples using anti-BRM (Fig. 6b) and anti-AR (Fig. 6c) antibodies.

In three cases, the positive staining for AR was detected in ACC cancer cells, indicative of AR ectopic expression in the ACC (Fig. 6c). The subsequent comparative analysis indicated that the expression of *SMARCA2* and *SMARCB1* genes negatively correlated with the expression of AR gene in the ACC samples (Fig. 6d, e).

### BRM acts mostly as transcriptional repressor in ACC

**Fig. 7:**
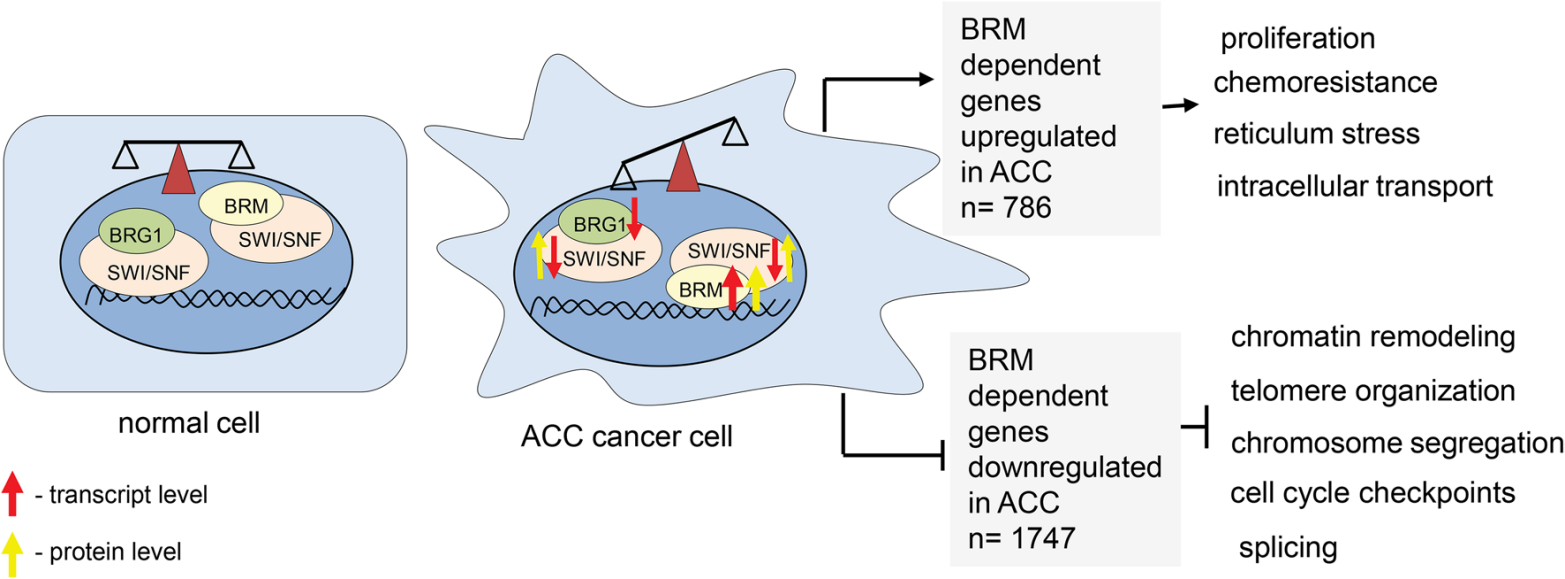
ACC is featured by affected balance between various classes of SWI/SNF CRCs

The analysis of genes directly targeted by BRM and upregulated in ACC displayed the 786 common genes. Among them the Gene Ontology analysis revealed the enrichment of cancer growth-promoting processes such as proliferation, intracellular transport, response to stress and chemoresistance. Interestingly, the 1747 genes were found to be BRM dependent and downregulated in adenoid cystic carcinoma cells. The subsequent Gene Ontology analysis identified the following processes: chromatin remodeling, telomere organization, chromosome segregation and cell cycle checkpoint as dependent on BRM and downregulated in ACC (Fig. 7, Supplementary Table 1 Sub-table 3 and 4) suggesting that BRM acts mostly as transcriptional repressor in ACC cells.

## Discussion

Surgery with postoperative radiotherapy remains the most effective therapeutic modality in patients with early-stage head and neck adenoid cystic carcinoma (Coca-Pelaz et al. 2015). In the case when surgery is impossible, the radiotherapy has been used and some attempts with chemotherapy including metronomic therapy; however, the outcome of such treatments is not satisfactory (Visa et al. 2010; Caballero et al. 2013; Ko et al. 2016; Cassidy et al. 2018). As there is no efficient therapy for the advanced stage of the adenoid cystic carcinoma there was an urgent need to investigate molecular basis of this type of cancer. Therefore, we performed the transcript profiling analysis of nine ACC samples and three normal salivary glands from publicly available datasets. We found that more than 8600 genes were mis-regulated in ACC samples compared to normal salivary gland tissue. 4044 of them were upregulated and 4603 downregulated. Interestingly, the genes with downregulated expression in ACC samples were related to chromatin or chromatin involving processes such as chromatin remodeling and organization, histone modifications, telomere maintenance as well as RNA splicing. This observation is in line with previous findings demonstrating that ACC is featured by mutations in genes coding for regulators of chromatin state including *SMARCA2*, a gene encoding BRM, the central ATPase of SWI/SNF ATP-dependent chromatin remodeling complex (Ho et al. 2013). The closer inspection of SWI/SNF CRCs revealed the mis-regulated expression of genes encoding its main subunits. Interestingly, almost all genes coding for core SWI/SNF subunits (*SMARCA4, SMARCB1, SMARCC1, SMARCC2*) as well as *ARID1* and *PBRM1* genes coding for auxiliary SWI/SNF subunits were downregulated. Surprisingly, the expression of *SMARCA2* gene encoding BRM ATPase was more than threefold upregulated.

As the SWI/SNF complex is composed of several subunits with defined stoichiometry, the protein level analysis of SWI/SNF subunits in ACC samples was performed. The IHC analysis confirmed the general overexpression of BRM protein in the most of the ACC cells compared to normal salivary gland tissues. In our study, we observed sporadically only some BRM-unstained cells but not BRM-unstained regions of ACC suggesting that the BRM protein accumulation is a general feature of ACC. The other SWI/SNF core complex subunits, namely INI1, BAF155 and BAF170 were also overexpressed in ACC at the protein level; however, the transcript levels of genes encoding these proteins were significantly downregulated in ACC cancer samples. These findings strongly suggest that core SWI/SNF complex subunits are precisely regulated at the protein level. Our findings suggest that in the case of adenoid cystic carcinoma the balance between classes of SWI/SNF complexes is shifted to the increased amount of BRM-containing rather than BRG1-containing complexes. Therefore, the proper control of gene expression may be affected in ACC due to the alterations in the balance between various classes of SWI/SNF complexes. This could lead to the aberrant expression of various genes involved in cell cycle control, proliferation and cell adhesion as these genes were found as targeted directly by BRM (Fig. 7). Additionally, the *SMARCA2* gene (coding for BRM) was found to be mutated in 5% of ACC samples. Interestingly, all mutations were located in the helicase domain of BRM protein (Ho et al. 2013) suggesting functional impairment of the mutated protein in ACC.

The SWI/SNF complex is involved in steroid hormone action and directly interacts with various steroid hormone receptors including androgen receptor (Sarnowska et al. 2016). The androgen receptor pathway was found as one of the most deregulated, with wide spectrum of AR transcript level in ACC patient samples. There was around ninefold difference between the highest and lowest AR transcript level in patient samples. It has been shown that AR overexpression is most frequently associated with salivary duct carcinomas. In several studies, the AR positivity was shown in 64–77% of all salivary duct carcinoma and 5% of ACC cases (Dalin et al. 2017). The other study showed AR abundance in about 6.6% cases of salivary gland adenoid cystic carcinoma (Ito et al. 2009). A number of patients with AR-positive salivary gland carcinoma have been treated with anti-androgen therapy. The treatment was well tolerated, but relapse was commonly observed. A 3-year progression-free survival was 12%, and a 5-year overall survival of 19% (Dalin et al. 2017).

Current clinical data indicate that the stage of ACC is the most significant factor influencing treatment outcome, whereas histological grade has no prognostic impact. Additionally, negative impact of vascular and neural infiltration on survival in male patients was shown (Spiro 1997). The disease is slightly more common in women (van der Wal et al. 2002); however, in our study, the number of women and men was equal. No factor has yet been shown to be responsible for the increased incidence of adenoid cystic carcinoma in female patients; however, its occurrence may be connected to hormones, especially that estrogen enhances the malignancy in ACC cells (Sumida et al. 2016).

In our study, we detected AR nuclear immunoreactivity in some examined cases. Noteworthy, the staining was observed in particular cancer cells only. Moreover, the statistically significant negative correlation between AR and *SMARCA2*(BRM) and *SMARCB1*(INI1) expression was found, suggesting transcriptional co-regulation between these genes, although further examination of the role of this interdependence needs to be performed. Thus, results presented here confirmed the hypothesis that ACC belongs to the most heterogeneous types of cancer and likely, therefore, the development of commonly used targeted therapy against this type of the disease is very difficult. Here we showed that the BRM protein is accumulating in nearly all ACC cells, therefore, representing the most homogeneous feature of ACC which has been so far discovered. Moreover, large clusters of both ACC down- and upregulated genes represent direct BRM targets. Among common ACC upregulated and BRM target genes, there were identified gene classes responsible for proliferation and chemoresistance processes, the two features strictly linked to the ACC aggressiveness. These findings are in line with the recently published association of BRM overexpression with the chemoresistance in ovarian cancer (Xu et al. 2018b). Given the observation that neither the regular chemotherapy nor metronomic one is sufficient for effective treatment of salivary gland ACC patients, the findings presented here may help to understand the molecular basis of the chemotherapy resistance in ACC cells and serve as solid basis for further investigations aiming to establish new, more effective forms of therapy.

## Conclusions

So far there is no targeted therapy for treatment of patients with adenoid cystic carcinoma in salivary gland. Previous study revealed ACC as one of the most heterogeneous cancers with strong aberrations in pathways connected to chromatin and DNA stability. Our results indicated that genes encoding subunits of SWI/SNF chromatin remodeling complex are missexpressed in ACC samples likely resulting in the disruption of the balance between classes of SWI/SNF complexes and finally leading to the global transcriptomic changes as well as resistance to chemotherapy. In summary, the findings presented here indicate an important role of the aberration of SWI/SNF CRCs in ACC development and chemoresistance and, therefore, may help to establish more efficient ACC-targeted therapy in the future.

## Electronic supplementary material

Below is the link to the electronic supplementary material.

Supplementary material 1 (XLSX 230 KB)

Supplementary material 2 (DOCX 24 KB)
