# Supplementary material for: Advanced adenoid cystic carcinoma (ACC) is featured by SWI/SNF chromatin remodeling complex aberrations
Source: J Cancer Res Clin Oncol. 2018 Oct 31;145(1):201–11. doi: 10.1007/s00432-018-2783-5 (PMC6326013; doi:10.1007/s00432-018-2783-5)
Supplement: Supplementary file 2 — Supplementary material 2 (DOCX 24 KB) [file 432_2018_2783_MOESM2_ESM.docx]

**Supplementary Table 2.** Pathways significantly deregulated in ACC samples.

| Pathway | Number of Entities | pValue |
| --- | --- | --- |
| Alpha6Beta4Integrin | 53 | 3,25E-05 |
| AndrogenReceptor | 98 | 1,18E-04 |
| BCR | 148 | 2,97E-04 |
| EGFR1 | 181 | 6,18E-13 |
| ID | 30 | 0,045792956 |
| IL2 | 72 | 1,38E-04 |
| IL3 | 76 | 0,001023341 |
| IL4 | 56 | 7,89E-05 |
| IL6 | 66 | 1,64E-04 |
| IL-7 | 16 | 0,015059053 |
| KitReceptor | 70 | 0,004207601 |
| NOTCH | 93 | 7,34E-09 |
| TCR | 140 | 7,15E-10 |
| TGFBR | 200 | 7,15E-10 |
| TNF alpha/NF-kB | 214 | 1,21E-05 |
| Wnt | 138 | 5,89E-04 |
